# Depression and anxiety among university students during the COVID-19 pandemic in Bangladesh: A web-based cross-sectional survey

**DOI:** 10.1371/journal.pone.0238162

**Published:** 2020-08-26

**Authors:** Md. Akhtarul Islam, Sutapa Dey Barna, Hasin Raihan, Md. Nafiul Alam Khan, Md. Tanvir Hossain

**Affiliations:** 1 Statistics Discipline, Science, Engineering and Technology (SET) School, Khulna University, Khulna, Bangladesh; 2 Sociology Discipline, Social Science School, Khulna University, Khulna, Bangladesh; Qazvin University of Medical Sciences, ISLAMIC REPUBLIC OF IRAN

## Abstract

The purpose of this study was to investigate the prevalence of depression and anxiety among Bangladeshi university students during the COVID-19 pandemic. It also aimed at identifying the determinants of depression and anxiety. A total of 476 university students living in Bangladesh participated in this cross-sectional web-based survey. A standardized e-questionnaire was generated using the Google Form, and the link was shared through social media—Facebook. The information was analyzed in three consecutive levels, such as univariate, bivariate, and multivariate analysis. Students were experiencing heightened depression and anxiety. Around 15% of the students reportedly had moderately severe depression, whereas 18.1% were severely suffering from anxiety. The binary logistic regression suggests that older students have greater depression (OR = 2.886, 95% CI = 0.961–8.669). It is also evident that students who provided private tuition in the pre-pandemic period had depression (OR = 1.199, 95% CI = 0.736–1.952). It is expected that both the government and universities could work together to fix the academic delays and financial problems to reduce depression and anxiety among university students.

## Introduction

The outbreak of coronavirus diseases (COVID-19) has been substantially influencing the life and living of people across the world, especially after the declaration of a global pandemic by the World Health Organization in the second week of March 2020 [1]. As of June 7, 2020, around 6.91 million people were infected with the COVID-19, with a confirmed fatality of another 0.4 million worldwide [2]. Hence, many countries implemented a range of anti-epidemic measures, such as restricting travel for foreign nationals [3], closing down public spaces, and shutting down the entire transit system [4, 5], to contain the transmission of the highly contagious infections from human-to-human.

Following the detection of first COVID-19 case on March 8, 2020 [6], Bangladesh like many other countries put the lockdown strategy into effect on March 26, 2020, to ensure ‘social distance’ through ‘home quarantine’ to curb the ‘spread’ among its population [7–9], since a precise treatment or vaccine for the infected and people at risk are yet to achieved by the global health community [10, 11]. However, all education institutions were closed initially from March 18 to March 31, 2020 across the country and later extended to the mid of June 2020 in phases [12, 13].

This unprecedented experience of ‘home quarantine’ under lockdown with the uncertainty of academic and professional career has multifaceted impacts on the mental health of students. For example, a Canadian study focusing on the effects of quarantine after the severe acute respiratory syndrome (SARS) epidemic found an association between longer duration of quarantine with a high prevalence of anxiety and depression among people [14]. The ongoing COVID-19 pandemic is creating a psycho-emotional chaotic situation as countries have been reporting a sharp rise of mental health problems, including anxiety, depression, stress, sleep disorder as well as fear, among its citizens [15–19], that eventually increased the substance use [15] and sometimes suicidal behavior [20–22]. Researchers in China observed that the greater exposure to ‘misinformation’ through social media are more likely contributing to the development of anxiety, depression, and other mental health problems among its population of different socioeconomic background [23–26]. Studies before the COVID-19 pandemic also suggested an inverse relationship between media exposure and mental health [27, 28]. On the contrary, a study in South Korea during the Middle East respiratory syndrome (MERS) reported a positive relationship between risk perception and media exposure [29].

Given the unexpected circumstances, it is crucial to explore the psycho-social experience of university students in Bangladesh, especially during the COVID-19 pandemic. Such a study is expected to measure the psychological impacts of an unforeseen emergency on students, as well as to formulate and execute effective interventions and strategies to mitigate the mental health of people at large. This study was designed to address the psychological problems experienced by university students in Bangladesh.

## Materials and methods

### Data source

**Fig 1:**
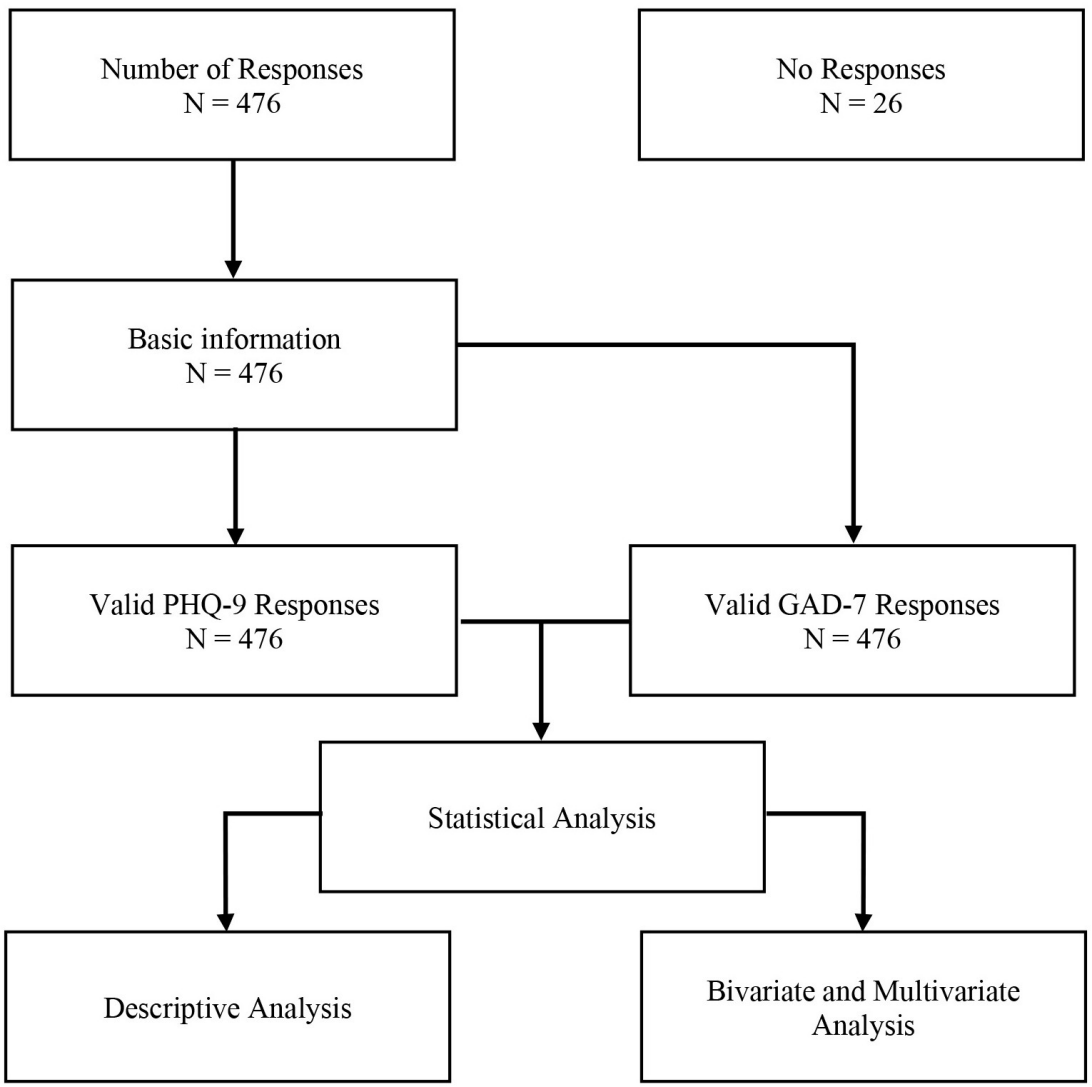
Flow chart.

The survey was conducted in the second week of May, from May 6 to May 12, 2020. Students enrolled in different universities across Bangladesh were the target population. An easy to understand questionnaire was used to collect ‘basic information,’ ‘depression,’ and ‘anxiety’ related information. An online-based platform was used to distribute the e-questionnaire, developed by using the Google Form, to the students. University students from all the divisions in Bangladesh were contacted through different social networks and interviewed (see Fig 1).

### Sampling technique

The Snowball sampling technique was used for collecting information from students. An informed consent form was attached to the e-questionnaire, and each participant consented to participate in the survey after reading the consent form. The participants were asked to share the e-questionnaire with their friends using their personal and institutional Facebook and Messenger.

### Ethical issues

This study was formally approved by the Ethical Clearance Committee of Khulna University, Bangladesh. The participants responded anonymously to the online survey by filling up an informed consent letter in the first section of the e-questionnaire. In the consent form, all the participants were provided with information concerning the research purpose, confidentiality of information, and right to revoke the participation without prior justification.

### Measures

#### Basic information

‘Basic Information’ contained the personal information of the respondents. Current ‘age’ of students (‘17–20’, ‘21–24’, ‘>24’), whether the student is ‘lagging behind study’ (‘yes’ and ‘no’), doing any sorts of ‘exercise during lockdown’ (‘yes’ and ‘no’), students who did ‘tuition’ before lockdown (‘yes’ and ‘no’), the gender of the student (‘male’ and ‘female’), ‘place of residence’ of students (‘rural’ and ‘urban’), is he/she ‘living with family’ during lockdown (‘yes’ and ‘no’).

#### Depression

Depression was determined by using the Patient Health Questionnaire (PHQ-9). PHQ-9 is an easy way to use in a questionnaire for screening depression of the responses that are used to predict depression of an individual and what state he/she is in during the survey. The scores in PHQ-9 range from ‘0 = not at all’ to ‘3 = nearly every day’ [30]. The reason for choosing PHQ-9 was that it proved to be a useful tool for detecting depression [31]. The levels of depression for the study were categorized as ‘mild = 5–9’, ‘moderate = 10–14,’ ‘moderately severe = 15–19,’ ‘severe = ≥ 20.’

#### Anxiety

Anxiety was evaluated by using the Generalized Anxiety Disorder (GAD-7). In the questionnaire, the questions were used for screening anxiety state of an individual on a scale ranging from ‘0 = not at all sure’ to ‘3 = nearly every day’ [32]. GAD-7 has been found successful in identifying anxiety among different populations and thus used for its reliability [33]. The levels of anxiety for the study were categorized as ‘none-minimal = <5,’ ‘mild = 5–9,’ ‘moderate = 10–14 and ‘severe = ≥ 15.’.

### Statistical analysis

Frequency tabulation was used to summarize basic information of respondents, as well as their response to depression and anxiety. Binary logistic regression [34] was used to identify variables influencing depression and anxiety among students by categorizing the outcome variable into two categories, i.e., depressed = ‘yes’ and ‘no’ and anxious = ‘yes’ and ‘no,’ which would provide a clearer idea about how intensely different factors are influencing the outcomes. Logistic regression generates the coefficients (and its standard errors and significance levels) of a formula to predict a logit transformation of the probability of the presence of the characteristic of interest:

$$logit\;(p)=b_{0}+b_{1}X_{1}+b_{2}X_{2}+\ldots +b_{k}X_{k}$$ (1)

Where *p* is the probability of the presence of the characteristic of interest. The logit transformation was defined as the logged odds:

$$Odds=\frac{p}{1-p}=\frac{Probality\;of\;presence\;of\;characteristic}{Probality\;of\;absence\;of\;charecteristic}$$ (2)

And,

$$logit\;(p)=loglog(\frac{p}{1-p})$$ (3)

Rather than choosing parameters that minimize the sum of squared errors (like in ordinary regression), estimation in logistic regression accepts parameters that maximize the likelihood of observing the sample values.

## Results

Table 1 shows the descriptive information of different selected variables of the university student in Bangladesh. Results show that 392 (82.4%) students were found to have mild to severe depressive symptoms, and 389 (87.7%) students were found to have mild to severe anxiety symptoms. More than 60% of the students were male (67.2%), and the rest were female. One in three students lived in rural areas (35.1%). Less than a quarter percent of students (24.8%) believed that they were not academically lagging, and just over 30% reportedly have exercise regularly during the lockdown at home.

**Table 1:** Frequency table for different selected variables.

Variables	Frequency	Percent
Age		
17–20	115	24.2
21–24	319	67.0
>24	42	8.8
Exercise		
No	293	61.6
Yes	183	38.4
Lagging academically		
No	118	24.8
Yes	358	75.2
Providing private tuition		
No	240	50.4
Yes	236	49.6
Gender		
Male	320	67.2
Female	156	32.8
Place of residence		
Rural	167	35.1
Urban	309	64.9
Living with family		
No	14	2.9
Yes	462	97.1
Depression		
None-minimal (<5)	84	17.6
Mild (5–9)	136	28.6
Moderate (10–14)	133	27.9
Moderately severe (15–19)	72	15.1
Severe (≥20)	51	10.7
Anxiety		
Minimal (<5)	87	18.3
Mild (5–9)	185	38.9
Moderate (10–14)	118	24.8
Severe (≥15)	86	18.1

Table 2 shows the prevalence of depression and anxiety among Bangladeshi university students. Out of the total 476 valid participants, 392 (82.4%) were found to have mild to severe depressive symptoms. Male (67.35%) had higher depressive symptoms than the female (32.65%) counterparts, whereas students in the early twenties (66.07%) showed higher depressive symptoms than other age groups. Depression was also prevalent among students with no physical exercise (62.24%) and those who consider themselves lagging behind others in terms of academic activities (76.78%). Besides, students living with families (96.93%) and in urban areas (65.05%) showed higher depressive symptoms. In the case of anxiety, 389 (87.7%) students exhibited mild to severe anxiety symptoms. Out of the total students suffering from an anxiety disorder, females (33.67%) had lower anxiety symptoms than males (66.33%), whereas students in the early twenties (66.58%) showed higher anxiety. Like depression, anxiety was also prevalent mostly among students with no physical exercise (61.95%), troubled with the thought of lagging behind others academically (76.60%). Moreover, students living in urban areas (62.21%) with families (96.40%) also showed symptoms of anxiety.

**Table 2:** Results of the PHQ-9 and GAD-7 (n = 476).

Variable	Depression					Anxiety			
	None-minimal	Mild	Moderate	Moderately severe	Severe	Minimal	Mild	Moderate	Severe
Gender									
Male	56 (11.8)	103 (21.6)	84 (17.6)	45 (9.5)	32 (6.7)	62 (13.0)	125 (26.3)	82 (17.2)	51 (10.7)
Female	28 (5.9)	33 (6.9)	49 (10.3)	27 (5.7)	19 (4.0)	25 (5.3)	60 (12.6)	36 (7.6)	35 (7.4)
Age									
17–20	20 (4.2)	39 (8.2)	27 (5.7)	19 (4.0)	10 (2.1)	20 (4.2)	53 (11.1)	25 (5.3)	17 (3.6)
21–24	60 (12.6)	86 (18.1)	94 (19.7)	44 (9.2)	35 (7.4)	60 (12.6)	113 (23.7)	85 (17.9)	61 (12.8)
>24	4 (0.8)	11 (2.3)	12 (2.5)	9 (1.9)	6 (1.3)	7 (1.5)	19 (4.0)	8 (1.7)	8 (1.7)
Exercise									
No	49 (10.3)	79 (16.6)	81 (17)	52 (10.9)	32 (6.7)	52 (10.9)	110 (23.1)	72 (15.1)	59 (12.4)
Yes	35 (7.4)	57 (12.0)	52 (10.9)	20 (4.2)	19 (4.0)	35 (7.4)	75 (15.8)	46 (9.7)	27 (5.7)
Lagging academically									
No	27 (5.7)	33 (6.9)	36 (7.6)	15 (3.2)	7 (1.5)	27 (5.7)	50 (10.5)	31 (6.5)	10 (2.1)
Yes	57 (12)	103 (21.6)	97 (20.4)	57 (12.0)	44 (9.2)	60 (12.6)	135 (28.4)	87 (18.3)	76 (16.0)
Providing private tuition									
No	44 (9.2)	71 (14.9)	59 (12.4)	37 (7.8)	29 (6.1)	47 (9.9)	91 (19.1)	57 (12.0)	45 (9.5)
Yes	44 (8.4)	65 (13.7)	74 (15.5)	35 (7.4)	22 (4.6)	40 (8.4)	94 (19.7)	61 (12.8)	41 (8.6)
Living with family									
No	2 (0.4)	4 (0.8)	3 (0.6)	1 (0.2)	4 (0.8)	0 (0.0)	8 (1.7)	1 (0.2)	5 (1.1)
Yes	82 (17.2)	132 (27.7)	130 (27.3)	71 (14.9)	47 (9.9)	87 (18.3)	177 (37.2)	117 (24.6)	81 (17.0)
Place of residence									
Rural	30 (6.3)	51 (10.7)	53 (11.1)	19 (4.0)	14 (2.9)	29 (6.1)	75 (15.8)	37 (7.8)	26 (5.5)
Urban	54 (11.3)	85 (17.9)	80 (16.8)	53 (11.1)	37 (7.8)	58 (12.2)	110 (23.1)	81 (17.0)	60 (12.6)

Table 3 reveals that students who thought that s/he was lagging behind others in academic activities were 1.8 times (95% CI: 1.098, 2.935) more likely to be depressed than the student with no such worries. Students living with families were 2.6 times (95% CI: 1.418, 4.751), more likely to be depressed than the students living apart from families. On the other hand, students providing supplementary classes before lockdown were 1.4 times (95% CI: 0.856, 2.227), more likely to show mild to severe anxiety symptoms than their counterparts with no such involvement. Students who were worried about their academic activities were 1.8 times (95% CI: 1.099, 2.883) more likely to exhibit mild to severe anxiety symptoms than students with no such worries. Students living with families were 1.8 times (95% CI: 1.021, 3.308), more likely to have mild to severe anxiety symptoms than students staying away from families during the lockdown.

**Table 3:** Binary logistic regression model predicting depressive and anxiety symptoms, based on the PHQ-9 and the GAD-7 scales.

Variables	Depression				Anxiety			
	*B*	P value	OR	95% CI Lower-Upper	*B*	P value	OR	95% CI Lower-Upper
Age								
17–20 ^ref^	1.000				1.000			
21–24	0.057	0.082	1.059	0.632–1.775	0.112	0.668	1.118	0.672–1.861
> 24	1.060	0.059	2.886	0.961–8.669	0.486	0.290	1.626	0.661–3.998
Providing private tuition								
No ^ref^	1.000				1.000			
Yes	0.181	0.046	1.199	0.736–1.952	0.322	0.018	1.381	0.856–2.227
Gender								
Male ^ref^	1.000				1.000			
Female	0.032	0.902	1.033	0.616–1.732	0.380	0.155	1.462	0.866–2.468
Place of residence								
Rural ^ref^	1.000				1.000			
Urban	0.022	0.933	1.022	.618–1.690	0.168	0.512	1.183	0.716–1.953
Lagging academically								
No ^ref^	1.000				1.000			
Yes	0.585	0.020	1.795	1.098–2.935	0.577	0.019	1.780	1.099–2.883
Exercise								
No ^ref^	1.000				1.000			
Yes	-0.128	0.601	0.880	0.544–1.422	-0.008	0.974	0.992	0.617–1.594
Living with family								
No ^ref^	1.000				1.000			
Yes	0.954	0.002	2.595	1.418–4.751	0.609	0.042	1.838	1.021–3.308

^ref.^ Reference group;

^OR:^ Odd ratio;

^CI:^ Confidence interval

## Discussion

COVID-19 pandemic came out as the most devastating and challenging crisis for public health in the contemporary world. Apart from the soaring mortality rate, nations across the globe have also been suffering from a spike of the excruciating psychological outcomes, i.e., anxiety and depression among people of all ages. University students are no exception, as all the educational institutions are unprecedentedly closed for more than usual, and for Bangladesh, it is more than two months in a row. Such closure, in general, triggers a sense of uncertainty about academic and professional career among the educands and intensifies persistent mental health challenges among university students [33, 35, 36]. Given such circumstances, the main goal of this study was to investigate the prevalence of depression and anxiety among the Bangladeshi university students during the COVID-19 pandemic and to explore the factors influencing the presence of depression and anxiety disorder.

The findings of the web-based cross-sectional survey indicate that more than two-thirds of the students were experiencing mild to severe depression (82.4%) and anxiety (87.7%). Earlier studies in Bangladesh observed the presence of both depression and anxiety among students in higher academia. For example, a survey of medical students in 2015 suggested that more than 50% of students in medical colleges are suffering from depression (54.3%) and anxiety (64.8%) [37]. Another study, on university students excluding the freshmen, complemented the previous work and found that the prevalence rate of depression and anxiety was 52.2% and 58.1%, respectively [38]. Compared to the earlier studies, our study suggests that university students in Bangladesh are experiencing an unparalleled growth of depression and anxiety under the current global pandemic situation.

The results also suggest that the university students’ involvement in private tuition is a critical factor in understanding the increased prevalence of depression and anxiety among them. In Bangladesh, a significant number of students are involved in part-time jobs, such as private tuition, to finance their educational expenses, and sometimes to support their families, and their reliance on private tutoring as a part-time job is increasing gradually [39]. However, being unable to provide tuition under the lockdown situation means disruption of regular income and joblessness. The prolonged unemployment, together with financial insecurity, is the most significant stressors contributing to the increased rates of depression and anxiety among university students in Bangladesh. A study suggests that unemployment is significantly associated with mental and somatic disorders, which could limit the individuals’ chances for feelings of achievement, accomplishment, and satisfaction, and eventually lead to the impairment of psychological functioning [40]. Self-esteem could also be affected by the loss of work as studies found that lack of family support during unemployment adversely affects the mental well-being of individuals [41, 42].

Apparently, the sudden joblessness and financial insecurity are putting the university students in an unpleasant situation, affecting their socioeconomic and mental well-being [43]. It has been well accepted that living with families strongly generate reassurance among the individuals, therefore, reduce depression and anxiety. Because positive family environments often benefit the mental health of the vulnerable youth experiencing depression or anxiety [44]. However, this pandemic has brought extreme financial pressure on families. Most of the families have been suffering from unmanageable debts and a decline in income, thus, leaving the family members in a traumatized situation [45, 46]. University students, who used to earn and contribute to their families before lockdown, can hardly assist their parents in this crisis moment. The results of this study suggest that despite living with family, anxiety and depressive symptoms have been increasing among university students in Bangladesh mainly due to financial insecurity.

Universities in developed countries put strict health protocols into action, such as washing hand, using face-mask, advising ‘stay-home’ strategy when sick, to facilitate continuation of education in higher academia and later switched to campus-wide online learning [47, 48]. In Bangladesh, the protective interventions, such as wearing mask or using the personal protective equipment, are yet to be enforced largely due to limited supplies [49, 50], hence, the government opted to implement the country-wide lockdown. Approximately two-thirds of the students are getting depressed thinking they might be falling academically behind their contemporaries in other parts of the world during the prolonged closure of universities. They, however, reiterated that the online classes could not fulfill their requirements [51] and a significant percentage of the students are still out of the reach of the online class. In addition, their research projects and internships had to be ceased since they were instructed to leave the halls (dormitories for students) of their respective universities [3]. Not only that, the Covid-19 crisis also created a severe challenge of the global reversion for the graduates to accomplish their future academic and working goals [52]. Although university closures were intended to keep students safe, for many, these notions came out with different sets of mental health issues.

Meanwhile, a study reported that graduate students generally experience significant amounts of stress and anxiety, which also affects their usual behavior [53]. The results in this study stressed on the fact that the nation-wide lockdown in Bangladesh is going to cause a significant disruption in the academic programs and create a gap in both teaching and learning. The academic delays could have long-term impacts on the psychology of students as they are more likely to be graduated later than they have expected. In this regard, faculties, as well as university authorities, should stay connected with the students using social media platforms and motivate them to move forward together during this difficult time.

Apart from the issues mentioned above, this study found no significant differences between male and female students with relation to depression or anxiety, thus complement previous studies [36, 37, 54]. However, Egyptian research remarked that female university students are more likely to suffer anxiety and less prone to depression than male students [55]. The current study did not find any statistically significant association between the socio-demographic variables (including place of residence and exercise) with depression and anxiety. A few studies, on the contrary, reported a significant association between socio-demographic variables [37] and exercise [56] with depression and anxiety. A Malaysian study reported substantial differences concerning age and permanent residence with depression or anxiety, however, observed no significant association between some socio-demographic variables (including gender, ethnicity, study major, monthly family income) and the psychological problems [36].

## Strengths and limitations

The strengths and limitations of the current study are determined by several issues. The e-questionnaire allows to assess the prevalence of anxiety and depression among university students while maintaining the WHO recommended “social distance” during the COVID-19 pandemic, which otherwise would be impossible. Moreover, the data for the e-survey were collected by globally validated standardized tools for quantitative analysis. On the contrary, given the limited resources available and the time-sensitivity of the COVID-19 outbreak, the snowball sampling strategy was chosen instead of random samples. In this cross-sectional study, the identified factors are regarded as associated factors, which could be either be the causes or the results of depression or anxiety. Furthermore, due to ethical requirements on anonymity and confidentiality, the contact details of the respondents was not collected. However, the use of validated screening e-questionnaire was considered as a cost-effective approach to explore the situation in general, therefore, used in this study. Since the research methodology could not reach people with medically examined depression and anxiety symptoms, the provision of the results may not fully reflect the severity of depressive and anxiety symptoms among students. Another limitation of this study is not using the tools designed specifically for the COVID-19 pandemic, such as the coronavirus anxiety scale (CAS). Meanwhile, it would be ideal for conducting a prospective study on the same group of participants with tools developed especially for the COVID-19 pandemic after a period to provide a concrete finding and to facilitate the demand for a focused public health initiative.

## Conclusion

Despite some limitations, this study gives the first empirical evidence that a large percentage of Bangladeshi university students have been suffering from depression and anxiety symptoms during the ongoing pandemic. In addition to academic and professional uncertainty, financial insecurity is contributing to the rise of depression and anxiety among university students. To minimize the growing mental health problems, the government, along with the universities, should work together to deliver promptly and accurately economy-oriented psychological support to the university students. To ensure the continuous involvement of students in educational processes, the universities should initiate all-inclusive online-based educational programs to reach out the students living in remote areas with or without devices in association with internet-service providers by providing scholarship or student loan. Furthermore, parents should be encouraged, by providing pandemic response and recovery support from the government, to create a friendly and positive family environment for university students without imposing pressure on the future academic and working career.

## Supporting information

S1 Data (DOCX)

## References

[1] World Health Organization. WHO Director-General’s opening remarks at the media briefing on COVID-19–11 March 2020 Geneva, Switzerland: World Health Organization; 2020 [cited 2020 18 April]. https://www.who.int/dg/speeches/detail/who-director-general-s-opening-remarks-at-the-media-briefing-on-covid-19-11-march-2020.

[2] Coronavirus disease (COVID-19): Situation report—138 [Internet]. Geneva, Switzerland: World Health Organization; 2020 [cited 07 June 2020]. https://www.who.int/docs/default-source/coronaviruse/situation-reports/20200606-covid-19-sitrep-138.pdf?sfvrsn=c8abfb17_4

[3] Zhai Y, Du X. Mental health care for international Chinese students affected by the COVID-19 outbreak. The Lancet Psychiatry. 2020;7(4):e22 10.1016/S2215-0366(20)30089-4 32199511 PMC7103995

[4] Ahmed MZ, Ahmed O, Aibao Z, Hanbin S, Siyu L, Ahmad A. Epidemic of COVID-19 in China and associated psychological problems. Asian Journal of Psychiatry. 2020;51:102092 10.1016/j.ajp.2020.102092 32315963 PMC7194662

[5] Chen L, Yuan X. China’s ongoing battle against the coronavirus: Why did the lockdown strategy work well? Socio-Ecological Practice Research. 2020:1–6. 10.1007/s42532-020-00048-1 PMC7158267 38624348

[6] The Daily Star. First coronavirus cases confirmed Dhaka, Bangladesh: Mahfuz Anam; 2020 [cited 2020 10 March]. https://www.thedailystar.net/frontpage/news/first-coronavirus-cases-confirmed-1878160.

[7] Bhuiyan AKMI, Sakib N, Pakpour AH, Griffiths MD, Mamun MA. COVID-19-related suicides in Bangladesh due to lockdown and economic factors: Case study evidence from media reports. International Journal of Mental Health and Addiction. 2020 10.1007/s11469-020-00307-y 32427168 PMC7228428

[8] Banik R, Rahman M, Sikder T, Gozal D. COVID-19 in Bangladesh: Public awareness and insufficient health facility remain key challenges. Public health. 2020 10.1016/j.puhe.2020.04.037 PMC7203024 32428773

[9] Jahid AM. Coronavirus pandemic: 45 districts now under complete lockdown. The Daily Star. 2020 26 April, 2020.

[10] Dong L, Hu S, Gao J. Discovering drugs to treat coronavirus disease 2019 (COVID-19). Drug Discoveries & Therapeutics. 2020;14(1):58–60. 10.5582/ddt.2020.01012 32147628

[11] Bai Y, Yao L, Wei T, Tian F, Jin D-Y, Chen L, et al Presumed asymptomatic carrier transmission of COVID-19. JAMA. 2020;323(14):1406–7. 10.1001/jama.2020.2565 32083643 PMC7042844

[12] Dhaka Tribune. Education institutions to remain closed till May 30. Dhaka Tribune. 2020 5 May, 2020;Sect. Education.

[13] United News of Bangladesh. Educational institutions to remain shut till June 15 Dhaka, Bangladesh: Educational institutions to remain shut till June 15; 2020 [updated 28 May 2020; cited 2020 06 June]. https://unb.com.bd/category/Bangladesh/educational-institutions-to-remain-shut-till-june-15/52155.

[14] Hawryluck L, Gold WL, Robinson S, Pogorski S, Galea S, Styra R. SARS control and psychological effects of quarantine, Toronto, Canada. Emerg Infect Dis. 2004;10(7):1206–12. 15324539 10.3201/eid1007.030703 PMC3323345

[15] Gritsenko V, Skugarevsky O, Konstantinov V, Khamenka N, Marinova T, Reznik A, et al COVID 19 fear, stress, anxiety, and substance use among Russian and Belarusian university students. International Journal of Mental Health and Addiction. 2020 10.1007/s11469-020-00330-z PMC7241583 32837418

[16] Ahorsu DK, Imani V, Lin C-Y, Timpka T, Broström A, Updegraff JA, et al Associations between fear of COVID-19, mental health, and preventive behaviours across pregnant women and husbands: An actor-partner interdependence modelling. International Journal of Mental Health and Addiction. 2020:1–15. 10.1007/s11469-020-00340-x PMC7289236 32837427

[17] Savitsky B, Findling Y, Ereli A, Hendel T. Anxiety and coping strategies among nursing students during the covid-19 pandemic. Nurse Education in Practice. 2020:102809 10.1016/j.nepr.2020.102809. PMC7264940 32679465

[18] Roy D, Tripathy S, Kar SK, Sharma N, Verma SK, Kaushal V. Study of knowledge, attitude, anxiety & perceived mental healthcare need in Indian population during COVID-19 pandemic. Asian journal of psychiatry. 2020;51:102083-. 10.1016/j.ajp.2020.102083 . 32283510 PMC7139237

[19] Xiao H, Zhang Y, Kong D, Li S, Yang N. Social capital and sleep quality in individuals who self-isolated for 14 days during the coronavirus disease 2019 (COVID-19) outbreak in January 2020 in China. Med Sci Monit. 2020;26:e923921–e. 10.12659/MSM.923921 . 32194290 PMC7111105

[20] Mamun MA, Griffiths MD. First COVID-19 suicide case in Bangladesh due to fear of COVID-19 and xenophobia: Possible suicide prevention strategies. Asian Journal of Psychiatry. 2020;51:102073 10.1016/j.ajp.2020.102073 32278889 PMC7139250

[21] Goyal K, Chauhan P, Chhikara K, Gupta P, Singh MP. Fear of COVID 2019: First suicidal case in India! Asian journal of psychiatry. 2020;49:101989-. Epub 2020/02/27. 10.1016/j.ajp.2020.101989 . 32143142 PMC7130010

[22] Russia Today. Reims football club doctor commits suicide in quarantine, mentions Covid-19 in note Moscow, Russia: RIA Novosti; 2020 [cited 2020 06 April]. https://www.rt.com/sport/485043-reims-doctor-suicide-coronavirus/.

[23] Bao Y, Sun Y, Meng S, Shi J, Lu L. 2019-nCoV epidemic: address mental health care to empower society. The Lancet. 2020;395(10224):e37–e8. 10.1016/S0140-6736(20)30309-3 32043982 PMC7133594

[24] Cao W, Fang Z, Hou G, Han M, Xu X, Dong J, et al The psychological impact of the COVID-19 epidemic on college students in China. Psychiatry Research. 2020;287:112934 10.1016/j.psychres.2020.112934 32229390 PMC7102633

[25] Gao J, Zheng P, Jia Y, Chen H, Mao Y, Chen S, et al Mental health problems and social media exposure during COVID-19 outbreak. PLOS ONE. 2020;15(4):e0231924 10.1371/journal.pone.0231924 32298385 PMC7162477

[26] Chen IH, Chen CY, Pakpour AH, Griffiths MD, Lin CY. Internet-related behaviors and psychological distress among schoolchildren during COVID-19 school suspension. J Am Acad Child Adolesc Psychiatry. 2020 Epub 2020/07/03. 10.1016/j.jaac.2020.06.007 . 32615153 PMC7833594

[27] Chaturvedi SK. Covid-19, coronavirus and mental health rehabilitation at times of crisis. Journal of Psychosocial Rehabilitation and Mental Health. 2020;7(1):1–2. 10.1007/s40737-020-00162-z 32292688 PMC7114947

[28] Wasserman IM. The impact of epidemic, war, prohibition and media on suicide: United States, 1910–1920. Suicide and Life-Threatening Behavior. 1992;22(2):240–54. 1626335

[29] Choi D-H, Yoo W, Noh G-Y, Park K. The impact of social media on risk perceptions during the MERS outbreak in South Korea. Computers in Human Behavior. 2017;72:422–31. 10.1016/j.chb.2017.03.004 32288176 PMC7126097

[30] Kroenke K, Spitzer RL, Williams JB. The PHQ-9: validity of a brief depression severity measure. J Gen Intern Med. 2001;16(9):606–13. Epub 2001/09/15. 10.1046/j.1525-1497.2001.016009606.x . 11556941 PMC1495268

[31] Martin A, Rief W, Klaiberg A, Braehler E. Validity of the brief patient health questionnaire mood scale (PHQ-9) in the general population. Gen Hosp Psychiatry. 2006;28(1):71–7. Epub 2005/12/27. 10.1016/j.genhosppsych.2005.07.003 . 16377369

[32] Spitzer RL, Kroenke K, Williams JBW, Löwe B. A brief measure for assessing generalized anxiety disorder: The GAD-7. Archives of Internal Medicine. 2006;166(10):1092–7. 10.1001/archinte.166.10.1092 16717171

[33] Hossain S, Anjum A, Uddin ME, Rahman MA, Hossain MF. Impacts of socio-cultural environment and lifestyle factors on the psychological health of university students in Bangladesh: A longitudinal study. J Affect Disord. 2019;256:393–403. 10.1016/j.jad.2019.06.001. 31226611

[34] Hosmer DW, Lemeshow S. Applied logistic regression. 2nd ed New York: Wiley; 2000.

[35] Mei SL, Yu JX, He BW, Li JY. Psychological investigation of university students in a university in Jilin province. Med Soc. 2011;24(5):84–6.

[36] Shamsuddin K, Fadzil F, Ismail WSW, Shah SA, Omar K, Muhammad NA, et al Correlates of depression, anxiety and stress among Malaysian university students. Asian Journal of Psychiatry. 2013;6(4):318–23. 10.1016/j.ajp.2013.01.014. 23810140

[37] Alim S, Rabbani M, Karim E, Mullick M, Al Mamun A, Khan M. Assessment of depression, anxiety and stress among first year MBBS students of a public medical college, Bangladesh. Bangladesh Journal of Psychiatry. 2015;29(1):23–9.

[38] Mamun MA, Hossain MS, Griffiths MD. Mental health problems and associated predictors among Bangladeshi students. International Journal of Mental Health and Addiction. 2019 10.1007/s11469-019-00144-8

[39] Pallegedara A, Mottaleb KA. Patterns and determinants of private tutoring: The case of Bangladesh households. International Journal of Educational Development. 2018;59:43–50. 10.1016/j.ijedudev.2017.10.004.

[40] Linn MW, Sandifer R, Stein S. Effects of unemployment on mental and physical health. Am J Public Health. 1985;75(5):502–6. 10.2105/ajph.75.5.502 . 3985238 PMC1646287

[41] Kasl SV, Gore S, Cobb S. The experience of losing a job: reported changes in health, symptoms and illness behavior. Psychosom Med. 1975;37(2):106–22. Epub 1975/03/01. 10.1097/00006842-197503000-00002 . 1135358

[42] Gore J, Holmes K, Smith M, Southgate E, Albright J. Socioeconomic status and the career aspirations of Australian school students: Testing enduring assumptions. The Australian Educational Researcher. 2015;42(2):155–77. 10.1007/s13384-015-0172-5

[43] Blustein DL. The importance of work in an age of uncertainty: The eroding work experience in America. New York: Oxford University Press; 2019.

[44] van Harmelen A-L, Gibson JL, St Clair MC, Owens M, Brodbeck J, Dunn V, et al Friendships and family support reduce subsequent depressive symptoms in at-risk adolescents. PLOS ONE. 2016;11(5):e0153715 10.1371/journal.pone.0153715 27144447 PMC4856353

[45] Fegert JM, Vitiello B, Plener PL, Clemens V. Challenges and burden of the Coronavirus 2019 (COVID-19) pandemic for child and adolescent mental health: A narrative review to highlight clinical and research needs in the acute phase and the long return to normality. Child and Adolescent Psychiatry and Mental Health. 2020;14:20 10.1186/s13034-020-00329-3 32419840 PMC7216870

[46] Mamun MA, Ullah I. COVID-19 suicides in Pakistan, dying off not COVID-19 fear but poverty?–The forthcoming economic challenges for a developing country. Brain, Behavior, and Immunity. 2020;87:163–6. 10.1016/j.bbi.2020.05.028. PMC7212955 32407859

[47] Liguori E, Winkler C. From offline to online: Challenges and opportunities for entrepreneurship education following the COVID-19 pandemic. Entrepreneurship Education and Pedagogy. 2020:2515127420916738. 10.1177/2515127420916738

[48] Perrone MA, Youssefzadeh K, Serrano B, Limpisvasti O, Banffy M. The impact of COVID-19 on the sports medicine fellowship class of 2020. Orthopaedic Journal of Sports Medicine. 2020;8(7):2325967120939901 10.1177/2325967120939901 PMC7433378 32874996

[49] Hasan K. Easing Covid-19 lockdown: Will wearing face masks be safe enough? Dhaka Tribune. 2020 5 9, 2020;Sect. Health.

[50] Rahman SM. Covid-19: Use of face mask. The Financial Express. 2020 4 13, 2020;Sect. Views.

[51] Dhawan S. Online learning: A panacea in the time of COVID-19 crisis. Journal of Educational Technology Systems. 2020:0047239520934018. 10.1177/0047239520934018

[52] Sahu P. Closure of universities due to coronavirus disease 2019 (COVID-19): Impact on education and mental health of students and academic staff. Cureus. 2020;12(4):e7541–e. 10.7759/cureus.7541 . 32377489 PMC7198094

[53] Garcia-Williams AG, Moffitt L, Kaslow NJ. Mental health and suicidal behavior among graduate students. Academic Psychiatry. 2014;38(5):554–60. 10.1007/s40596-014-0041-y 24711096

[54] Tayefi B, Eftekhar M, Tayefi M, Darroudi S, Khalili N, Mottaghi A, et al Prevalence and socio-demographic correlates of mental health problems among Iranian health sciences students. Academic Psychiatry. 2020;44(1):73–7. 10.1007/s40596-019-01121-y 31625073

[55] Wolf MR, Rosenstock JB. Inadequate sleep and exercise associated with burnout and depression among medical students. Acad Psychiatry. 2017;41(2):174–9. Epub 2016/03/16. 10.1007/s40596-016-0526-y . 26976402

[56] Abdallah AR, Gabr HM. Depression, anxiety and stress among first year medical students in an Egyptian public university. International Research Journal of Medicine and Medical Sciences. 2014;2(1):11–9.

