# Supplementary material for: Depression and anxiety among university students during the COVID-19 pandemic in Bangladesh: A web-based cross-sectional survey
Source: PLoS One. 2020 Aug 26;15(8):e0238162. doi: 10.1371/journal.pone.0238162 (PMC7449469; doi:10.1371/journal.pone.0238162)
Supplement: S1 Data — (DOCX) [file pone.0238162.s001.docx]

We have published our data set to <https://dataverse.harvard.edu/dataset.xhtml?persistentId=doi%3A10.7910%2FDVN%2FN5BUJR>

Title: Data_Bangladesh_COVID-19

DOI: <https://doi.org/10.7910/DVN/N5BUJR>
